# Resistance to thyroid hormone due to defective thyroid receptor alpha

**DOI:** 10.1016/j.beem.2015.07.007

**Published:** 2015-08

**Authors:** Carla Moran, Krishna Chatterjee

**Affiliations:** Metabolic Research Laboratories, Wellcome Trust-MRC Institute of Metabolic Science, University of Cambridge and National Institute for Health Research Cambridge Biomedical Research Centre, Addenbrooke's Hospital, Cambridge, CB2 0QQ, UK

**Keywords:** resistance to thyroid hormone, thyroid receptor α, dominant negative inhibition, corepressor

## Abstract

Thyroid hormones act via nuclear receptors (TRα1, TRβ1, TRβ2) with differing tissue distribution; the role of α2 protein, derived from the same gene locus as TRα1, is unclear.

Resistance to thyroid hormone alpha (RTHα) is characterised by tissue-specific hypothyroidism associated with near-normal thyroid function tests. Clinical features include dysmorphic facies, skeletal dysplasia (macrocephaly, epiphyseal dysgenesis), growth retardation, constipation, dyspraxia and intellectual deficit. Biochemical abnormalities include low/low-normal T4 and high/high-normal T3 concentrations, a subnormal T4/T3 ratio, variably reduced reverse T3, raised muscle creatine kinase and mild anaemia.

The disorder is mediated by heterozygous, loss-of-function, mutations involving either TRα1 alone or both TRα1 and α2, with no discernible phenotype attributable to defective α2. Whole exome sequencing and diagnostic biomarkers may enable greater ascertainment of RTHα, which is important as thyroxine therapy reverses some metabolic abnormalities and improves growth, constipation, dyspraxia and wellbeing.

The genetic and phenotypic heterogeneity of RTHα and its optimal management remain to be elucidated.

## Introduction

The diverse physiological actions of thyroid hormones (TH: thyroxine, T4; triiodothyronine, T3) include regulation of growth, control of metabolic rate, positive chronotropic and inotropic cardiac effects and development of the central nervous system (Table 1). TH synthesis is controlled by hypothalamic thyrotropin-releasing hormone (TRH) and pituitary thyroid stimulating hormone (TSH) and, in turn, T4 and T3 regulate TRH and TSH synthesis as part of a negative feedback loop. These physiological effects are mediated by thyroid hormone-dependent changes in expression of specific target genes in different tissues (Table 1). The cellular entry of thyroid hormones , particularly in the central nervous system, is mediated by a membrane transporter [monocarboxylate transporter 8 (MCT8)] [1]. Intracellularly, deiodinase enzymes (DIOs) mediate hormone metabolism, with a high-affinity type 2 enzyme (DIO2) mediating T4 to T3 conversion in the central nervous system (CNS) including pituitary and hypothalamus, type I deiodinase (DIO1) generating T3 in peripheral tissues, and type 3 deiodinase (DIO3) mediating catabolism of thyroid hormones to inactive metabolites [2]. Thyroid hormones alter target gene expression via a receptor protein (TR), belonging to the steroid/nuclear receptor superfamily of ligand-inducible transcription factors. TR binds preferentially to regulatory DNA sequences (thyroid hormone response elements, TREs) in target gene promoters as a heterodimer with the retinoid X receptor (RXR), although the receptor can bind some TREs as a homodimer or monomer. In the absence of hormone, unliganded receptor homodimers/heterodimers recruit a protein complex containing corepressors (e.g. nuclear receptor corepressor [NCoR]; silencing mediator for retinoic acid and thyroid receptors [SMRT]) and histone deacetylase (HDAC) to repress basal gene transcription. Receptor occupancy by hormone (T3) results in dissociation of the corepressor complex and relief of repression together with recruitment of coactivator proteins which mediate transcriptional activation [3].

**Fig. 1:**
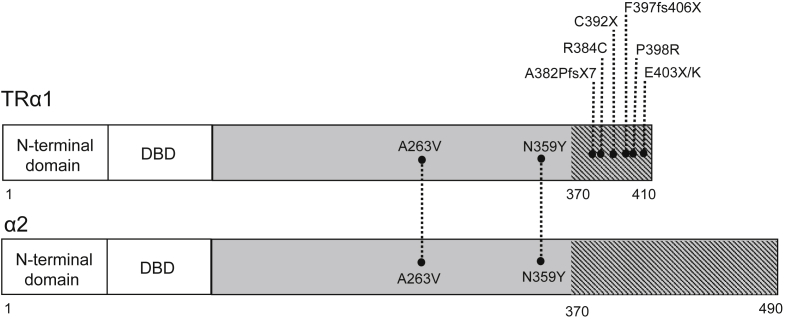
Schematic illustrating the domain structure of proteins derived from the sense strand of *THRA locus*, together with the location of known mutations. Thyroid hormone receptor α1 (TRα1) and the splice variant protein α2 aligned by their DNA binding domains (DBD), which are identical. The ligand binding domains are coloured in grey, with non homologous areas shaded. The location of each known TRα mutation is depicted; only A263V and N359Y affect both TRα1 and α2 transcripts; the remainder of the mutations only affect TRα1.

In humans, two highly homologous thyroid hormone receptors, TRα and TRβ are encoded by genes (*THRA*, *THRB*) on chromosomes 17 and 3, respectively. Two different proteins are generated from the *THRA* locus by alternate splicing: TRα1 is an ubiquitously expressed receptor isoform, with particular abundance in the central nervous system, myocardium, gastrointestinal tract and skeletal muscle; α2 protein, which exhibits a divergent carboxy-terminal region such that it is unable to bind thyroid hormones (Fig. 1), is expressed in a variety of tissues (e.g. brain and testis) and its biological function is poorly understood [4]. The REV-ERBα gene, located on the opposite strand of the *THRA* locus, is transcribed to generate a nuclear receptor which is involved in regulating circadian rhythm [5]. *THRB* generates two major receptor isoforms, TRβ1 and TRβ2, which differ in their amino-terminal regions; TRβ1, which is widely expressed, is the predominant isoform in liver and kidney, while TRβ2 expression is limited principally to the hypothalamus, pituitary, inner ear, and retina [4].

Resistance to Thyroid Hormone beta (RTHβ), a dominantly-inherited disorder due to *THRB* mutations, is readily recognized due to a characteristic biochemical signature of elevated circulating T4 and T3 with non-suppressed pituitary TSH levels reflecting central (hypothalamic–pituitary) refractoriness to thyroid hormone action and is associated with variable resistance to hormone action in peripheral tissues [6]. The incidence of RTHβ is ∼1 in 40,000, and several hundred heterozygous, β receptor mutations which localise to three hotspots or clusters within its ligand binding domain (LBD) have been identified in this disorder [7]. Consistent with its mode of inheritance, mutant β receptors in RTHβ inhibit the function of their wild type receptor counterparts in a dominant negative manner; constitutive target gene repression due to failure of corepressor complex dissociation from mutant TRβ represents a likely mechanism for such dominant negative inhibition [8].

Human TRβ and TRα exhibit marked aminoacid sequence similarity, including (80%) in their hormone binding domains; accordingly, with ∼160 different receptor mutations known to be associated with RTHβ, the identification of a homologous human disorder with defective TRα had been anticipated. Supporting this notion, murine transgenic models harbouring different, heterozygous, TRα mutations are viable and exhibit recognisable abnormalities, but with little perturbation of thyroid function [9–12]; such absence of an overt biochemical, thyroid, phenotype likely explains why the homologous human disorder had eluded discovery. However, human *THRA* mutations have now been identified in 14 cases from 10 different families, with hypothyroid features and thyroid hormone resistance in target tissues, but associated paradoxically with near-normal thyroid function tests [13–20]. Here, we review the clinical features, differential diagnosis, molecular genetics, pathogenesis and management of Resistance to Thyroid Hormone due to defective thyroid receptor alpha (RTHα).

## Clinical features

At birth, some features (e.g. macroglossia, poor feeding, hoarse cry), recognized in hypothyroidism, have been noted [16,17]. Several patients were investigated in infancy for growth retardation which in some cases predominantly affected the lower segment [13,18]. Abnormal physical characteristics in the majority of cases include macrocephaly, broad facies, hypertelorism, a flattened nose, prominent tongue and thick lips [13–18]; indeed, five cases were identified following genetic investigation of a clinic patient cohort with these shared characteristics [18]. An excessive number of skin tags and moles have been noted, particularly in adults [13,16,17].

### Biochemical

The most consistent pattern of thyroid function tests comprises low or low-normal free T4, and high or high-normal free T3, resulting in an abnormally low T4/T3 ratio; reverse T3 levels were subnormal in severe cases [13–17] but can be normal [19,20]. A mild, usually normocytic anaemia [13,15–18] with normal haematinics (Iron, B12, folate) and haemolytic indices (reticulocyte count, circulating haptoglobin and lactate dehydrogenase) [16] and raised muscle creatine kinase levels [13,15–17] are a consistent abnormality. Raised total and LDL cholesterol levels have been documented [15,16], even in childhood cases.

### Skeletal

Radiographic abnormalities in childhood include delayed fontanelle fusion and excessively serpiginous cranial sutures (“wormian bone” appearance), together with delayed dentition [13,14]; femoral epiphyseal dysgenesis was present in childhood [13,18] but not in adult life [16]; bone age can be delayed [13,14,18]. A thickened calvarium (skull vault) and cortical hyperostosis in long bones, together with increased bone mineral density, is present in most cases, especially adults.

### Neurocognitive

In childhood, patients showed delayed milestones (motor, speech). Slow initiation of motor movement, together with fine and gross motor incoordination, manifesting as dyspraxia or a broad-based, ataxic gait and slow, dysarthric speech were a consistent feature. Their IQ was variably reduced, being markedly subnormal, with seizures in one case [16].

### Gastrointestinal

Reduced frequency of bowel movements is a common finding, with severe constipation being a significant problem in several cases [13,16,18].

### Cardiovascular

Bradycardia [13,16,17] is typical, with abnormal sympathovagal balance and indices of cardiac contractility in the hypothyroid range [16].

### Metabolic & endocrine

Resting energy expenditure (metabolic rate) was low in most patients [13,16,17,19]. Both male and female to offspring transmission of TRα defects has been recorded [14,17,18], suggesting that fertility in either gender is not unduly compromised.

Table 2 summarises known clinical features of RTHα, together with clinical, biochemical and physiological investigations which can identify recognised abnormalities.

## Differential diagnosis

RTHα could be suspected in childhood patients with dysmorphic features or retardation of growth and psychomotor development or adults with a history of such features. Whilst a low ratio of circulating T4/T3 levels is a consistent feature which could identify potential cases, this biochemical abnormality is also a feature of disorders (genetic or environmental) with dyshormonogenetic hypothyroidism or Allan–Herndon–Dudley syndrome due to defects in the MCT8 gene. Table 3 shows clinical and biochemical features which could differentiate between these entities.

## Molecular genetics

Affected individuals are heterozygous for *THRA* mutations which occurred *de novo* in six cases [13,18–20] or were familial [14,17,18]. Hitherto, two broad classes of receptor defect have been identified: either highly deleterious, frameshift/premature stop mutations; or less severe, missense, aminoacid changes (Fig. 1). None of the mutations affect the REV-ERBα gene, transcribed from the opposite strand of the *THRA* locus.

**Fig. 2:**
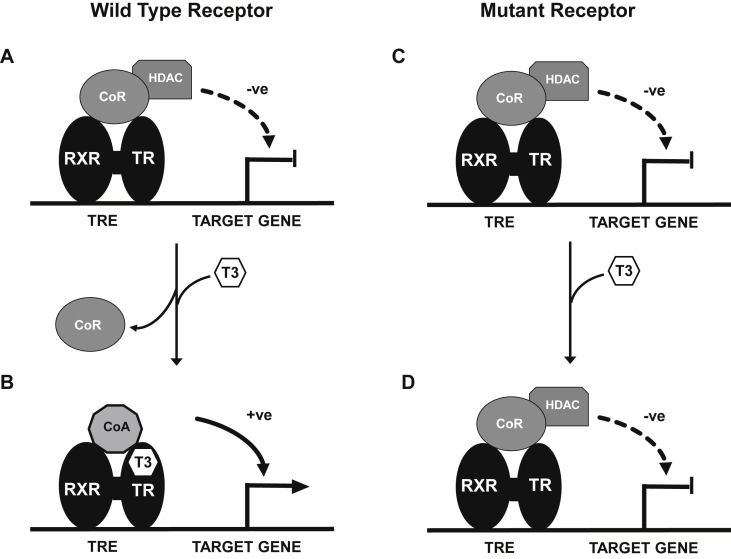
Model of transcriptional regulation of target genes by thyroid receptors (TR). Unliganded TRs [usually bound as a heterodimer with retinoid X receptor (RXR) to specific regulatory segments in the target gene (thyroid hormone response elements; TREs)] recruit a corepressor complex (CoR) including histone deacetylase (HDAC), which acts to inhibit gene transcription (Panel A). Receptor occupancy by T3 (Panel B) promotes dissociation of the corepressor complex and recruitment of a coactivator complex (CoA), mediating activation of target gene transcription. Mutant TRs can recruit the CoR complex and inhibit basal gene transcription (Panel C) but are unable to bind T3 and hence cannot release the CoR complex or recruit CoA, resulting in persistent inhibition of gene transcription, even in the presence of hormone (Panel D).

Most cases harbour mutations which selectively disrupt the carboxyterminal activation domain of TRα1 [13,14,17,18]. Consistent with this, where their functional properties have been elucidated, the mutant receptors fail to bind ligand and are devoid of transcriptional activity [13,15,16]. Similar to TRβ mutations in RTHβ, TRα1 mutants inhibit the function of their wild type receptor counterparts in a dominant negative manner when they are coexpressed [13,14,16]. As has been delineated in RTHβ, constitutive binding of mutant TR to corepressors, with failure of corepressor dissociation and coactivator recruitment following T3 occupancy, likely mediates dominant negative inhibition (Fig. 2). Expression of TH-responsive target genes in mutation-containing patient peripheral blood mononuclear cells is blunted, suggesting that such dominant negative inhibition also occurs *in vivo* [13,16,17,19].

In one family, three affected individuals harbored a missense mutation (A263V) in *THRA*, which affects both TRα1 and α2 proteins [17]. Furthermore, this aminoacid change in TRα1 is homologous to a TRβ mutation (A317V) recognized to mediate RTHβ, with that TRβ mutation localising to one of the mutation clusters within its ligand binding domain. The A263V TRα1 mutant was transcriptionally impaired at low T3 concentrations, but higher TH levels restored mutant receptor function and reversed its dominant negative inhibitory activity. In the α2 protein background, the A263V mutation exhibited no added gain or loss-of-function; this is consistent with the uncertain functional role of normal α2 and previous observations suggesting that it is unable to heterodimerise with RXR, bind TREs or exert dominant negative activity via corepressor recruitment [21–23]. Such absence of altered mutant α2 function correlated with the observation that patients with the combined TRα1 and α2 mutation had no discernible extra phenotypes, attributable to A263V mutant α2 [17].

A 27yr old female, harboring a *de novo* mutation (N359Y) affecting both TRα1 and α2 proteins, exhibited a low FT4/FT3 ratio but other features (micrognathia, clavicular agenesis, hypoplasia, metacarpal fusion and syndactyly of digits, hyperparathyroidism and chronic diarrhoea) that have not been recorded in other RTHα cases [19]. Studies showed impaired function and dominant negative activity of N359Y mutant TRα1, with some weakening of dominant negative activity of N359Y mutant α2, particularly when coexpressed with normal TRβ1. T3 treatment in the patient suppressed TSH and raised energy expenditure and SHBG levels; paradoxically, unlike other RTHα cases, her heart rate increased and diarrhoea worsened [19]. Although conventional and whole exome sequencing ruled out abnormalities in other candidate genes, it is not certain whether all the clinical features of this case are attributable solely to the N359Y *THRA* defect [24].

Whole genome sequencing in human autism spectrum disorder has identified a patient with a *de novo*, missense, variant (R384C) in TRα1 [20]. This aminoacid change is almost certainly pathogenic, being functionally deleterious when studied in the context of murine TRα1 [9]. Interestingly, transgenic mice harboring this mutation exhibit locomotor (ataxia) and behavioural abnormalities (anxiety, depression) which can be alleviated by thyroid hormone treatment initiated even in adulthood [25,26].

## Pathogenesis

Many clinical features in RTHα are typical of uncorrected hypothyroidism in childhood or adult life. Patent cranial sutures, delayed dentition, femoral epiphyseal dysgenesis (disordered, endochondral ossification) and wormian bones (disordered, intramembranous ossification) are recognized features of childhood thyroid hormone deficiency [27,28]; macrocephaly may reflect delayed fontanelle closure and hypothyroid facies includes a flattened nasal bridge; such skeletal dysplasia is associated with growth retardation (predominantly lower segmental) and delayed bone age in childhood or adult short stature. Similarly, diminished colonic motility resulting in slow-transit constipation with colonic dilatation (megacolon) or even ileus are reported in human hypothyroidism [29]. Skeletal abnormalities (growth retardation, delayed tooth eruption, patent cranial sutures, epiphyseal dysgenesis) and intestinal dysmotility in human RTHα are recapitulated in mutant TRα1 mutant mouse models [11,30].

Although borderline, the biochemical abnormalities found in RTHα cases (disproportionately raised/high-normal T3 and low/low-normal T4 levels, resulting in a markedly reduced T4/T3 ratio together with low rT3 levels in some cases) may reflect altered metabolism of thyroid hormones in these patients. One possibility is that, as has been documented in mice with a dominant negative TRα1 mutation (TRα1-PV) [10], increased hepatic DIO1 levels augment T4 to T3 conversion; alternatively, reduced tissue levels of DIO3, whose expression is TRα1 regulated [31], may contribute to these abnormalities with decreased inner-ring deiodination of T4 to rT3 and T3 to T2.

DIO3 is also expressed in skin and inhibition of the enzyme in this tissue enhances keratinocyte proliferation in mice [32]. Accordingly, it is tempting to speculate that cutaneous DIO3 deficiency in RTHα patients might, at least in part, mediate propensity to excess skin tags and moles.

Anaemia in RTHα patients correlates with documented abnormal erythropoiesis and reduced haematocrit in TRα null or mutant mice [33,34]. Normal haematinics in patients suggests defective proliferation or differentiation of erythroid progenitors, with the mechanism remaining to be elucidated.

Idiopathic epilepsy which was noted in one human case [16] correlates with heightened susceptibility to seizures following photic [11] or audiogenic [25] stimulation and aberrant development of GABAergic inhibitory interneurons [35] in mutant mice harbouring different TRα1 mutations.

**Fig. 3:**
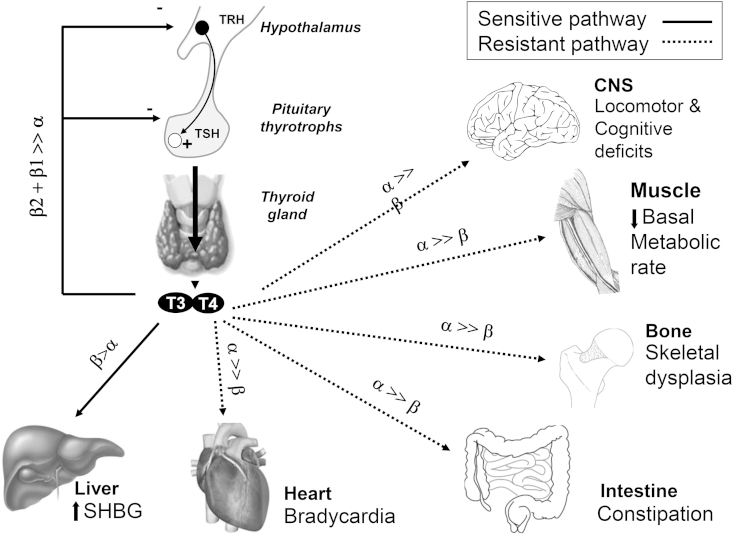
Summary of the major tissue actions of thyroid hormone, together with the receptor subtypes mediating these effects. In RTHα, tissues expressing mainly TRα would be resistant to thyroid hormone action with TRβ-expressing tissues being sensitive.

Following thyroxine treatment in physiological dosage, tissues of RTHα patients exhibit variable responses: thus, TSH levels suppress readily, implying preserved sensitivity within the hypothalamic–pituitary–thyroid axis; conversely, cardiac parameters, resting energy expenditure and muscle CK levels are less responsive [16,17]. Overall, these observations are consonant with thyroid hormone resistance in organs (e.g. myocardium, skeletal muscle, gastrointestinal tract) expressing predominantly TRα1, with preservation of TH sensitivity in TRβ-expressing tissues (hypothalamus, pituitary, liver) (Fig. 3).

## Treatment

Thyroxine therapy raises metabolic rate, serum IGF1 and SHBG and lowers elevated LDL cholesterol and muscle creatine kinase levels [13,15–17]; these changes may limit weight gain, especially in older patients. In the childhood case we first described [13], five years of thyroxine therapy has been clearly beneficial, improving overall height and subischial leg length, alleviating constipation (with associated restoration of contractile activity in colonic manometry) and improving wellbeing (Moran & Chatterjee, unpublished observations). Low-normal IGF1 levels prompted the addition of growth hormone to thyroxine therapy in another childhood case [15], but with little further improvement in growth. Treatment from early childhood in cases harbouring mutant TRα1 whose dysfunction is reversible at higher TH levels might have ameliorated their phenotype [17]. In adult life, these individuals report that thyroxine therapy improves dyspraxia and enhances social interaction (Moran & Chatterjee, unpublished observations). In contrast, in most cases, anaemia persists following thyroxine therapy; and, relative to the rise in TH levels, changes in cardiac parameters (e.g. heart rate, indices of myocardial contractility) are blunted [16,17].

Following thyroxine treatment, TSH levels suppress readily with elevation of FT3 to supraphysiologic levels; serum SHBG may rise further from high-normal baseline levels [13] and biochemical markers of bone turnover became progressively elevated in one case [16]. These observations raise the possibility that chronic, excess TH exposure in thyroxine-treated RTHα patients might lead to unwanted toxicities in normal TRβ-containing tissues. In this regard, future therapies which could be developed include TRα1-selective thyromimetics [36], to selectively activate either residual, normal TRα1 or partially defective, mutant TRα1 and overcome resistance in TRα-expressing tissues.

As described above, many *THRA* defects in RTHα abrogate hormone binding to receptor, such that dominant negative inhibition exerted by mutant TRα1 *in vitro* or in patient's cells studied *ex vivo* is irreversible, even following exposure to high T3 levels. Here, developing small molecules which either inhibit TR interaction with the corepressor complex or its histone deacetylase enzymatic activity, might represent a rational therapeutic approach. Supporting this notion, introduction of a mutation in NCoR that abrogates its interaction with TR [37] or administration of suberoylanilide hydroxamic acid, an inhibitor of histone deacetylase [38], ameliorates phenotypic abnormalities (growth, bone development) in the murine TRα1-PV mutant model of RTHα.

## Summary and conclusions

RTHα, a dominantly-inherited or sporadic disorder, due to heterozygous *THRA* mutations affecting TRα1 alone or in combination with variant α2 protein, is characterised by clinical, biochemical and physiological features of hypothyroidism in specific tissues, together with subtle abnormalities (low T4/T3 ratio, variably reduced rT3) of thyroid function. Preliminary experience suggests that thyroxine therapy is beneficial.

Given the estimated prevalence (∼1 in 40,000) of RTHβ, with over 160 different TRβ mutations being recorded hitherto, it is highly likely that RTHα is more common but not fully ascertained, either because the disorder lacks a clearcut, diagnostic signature of biochemical abnormalities or is associated with unexpected phenotypes (e.g. autism spectrum disorder). In this context, it is interesting to note that interrogation of databases (e.g. ExAC, 60,000 Exomes) reveals at least 101 non synonymous variants in *THRA* (52 common to TRα1/α2; 3 TRα1-specific; 49 α2-specific); at least five variants are potentially damaging, with aminoacid changes in codons that are homologous to residues in TRβ known to be mutated in association with RTHβ (http://exac.broadinstitute.org/gene/ENSG00000126351).

The discovery of additional biomarkers in RTHα would be useful. Specifically, the discovery of a combination of abnormal metabolites and/or proteins which can constitute a specific diagnostic test, would enable more complete ascertainment of the disorder, with earlier commencement of TH treatment in cases being potentially more effective. Furthermore, during TH therapy, markers which better indicate correction of resistance in TRα-expressing tissues or toxicity in TRβ-containing organs would be of utility.

**Practice points**

• Growth retardation, macrocephaly, skeletal dysplasia and constipation are common clinical findings in TRα-mediated Resistance to thyroid hormone (RTHα).
• Biochemical abnormalities include low T4/T3 ratio, subnormal reverse T3, raised muscle creatine kinase and anaemia.
• Thyroxine therapy reverses hypothyroidism in hormone-resistant TRα target tissues and is of symptomatic benefit. However, careful monitoring for adverse sequelae of excessive TH exposure in hormone-sensitive TRβ tissues, is warranted.

**Research agenda**

• Is RTHα more prevalent than currently known and could it be associated with unexpected clinical phenotypes?
• Can circulating biomarkers, which enable specific diagnosis of the disorder or guide TH therapy, including preventing unwanted toxicity in TRβ-expressing tissues, be developed?
• Can hormone resistance and dominant negative inhibition in selected target tissues be modelled in mutation-containing, patient-derived cells (either primary or derivatives of inducible pluripotent stem cells) studied *ex vivo*?
• Can TRα1 isoform-selective agonists be developed. Alternatively can transcriptional repression by mutant TRα1 be relieved by developing agents which either dissociate mutant receptor from the corepressor complex or inhibit its histone deacetylase activity?
• Can earlier (possibly antenatal) diagnosis, together with therapeutic intervention, prevent the skeletal and neurocognitive deficits in this disorder?

## Disclosures

None of the authors have anything to disclose.

## Figures and Tables

**Table 1:** Summary of some major physiological actions of thyroid hormone in tissues and associated target genes.

Actions of thyroid hormone		
Tissue	Action	Target genes
Brain	Cortical & cerebellar development; myelination	Krüppel-like factor 9; Hairless; Myelin basic protein
Liver	Lower cholesterol	LDL receptor
Raises SHBG	SHBG
Myocardium	Positive inotropic and chronotropic effect	α- myosin heavy chain Sarcoplasmic Ca^2+^-ATPase
Hypothalamus	Inhibits TRH secretion	Pro-thyrotrophin releasing hormone
Pituitary	Inhibits TSH secretion	TSH α and β subunits
Multiple	Increases basal metabolic rate	Multiple

**Table 2:** Summary of clinical features and suggested investigations for resistance to thyroid hormone alpha.

System	Clinical feature/phenotype	Investigations and possible findings
Appearance	• Flattened nasal bridge • Broad face, thickened lips^a^ • Macroglossia^a^ • Coarse facies, skin tags and moles^a^	○ *Dysmorphology*
Skeletal	• Disproportionate short stature^a^ • Macrocephaly^a^ • Delayed tooth eruption^a^	○ *Auxology*: reduced total height, normal sitting height but reduced subischial leg length, increased head circumference for age (children) or height (adults). Weight or BMI may be increased. ○ *Skull radiograph*: thickened calvarium, delayed fontanelle fusion,^b^ excessively serpiginous lambdoid suture (Wormian bones)^b^ ○ *Pelvic and long bone radiographs*: Cortical hyperostosis, femoral epiphyseal dysgenesis^b^ ○ *Spine radiograph*: Scalloped vertebral bodies ○ *Dental radiograph*: Delayed tooth eruption ○ *Bone age radiograph*: Delayed carpal bone maturation^b^ ○ *DXA scan or quantitative CT*: increased bone mineral density at hip
Gastrointestinal	• Constipation^a^	○ *Abdominal radiograph*: dilated bowel loops and impacted faecal matter ○ *Colonic manometry:* reduced peristalsis
Cardiovascular	• Bradycardia • Low blood pressure for age and gender	○ *Cardiac telemetry*: reduced average sleeping heart rate ○ *Spectral analysis of cardiac autonomic tone*: increased parasympathetic (vagal) tone ○ *Echocardiography*: hypothyroid indices of contractility
Metabolic	• Low metabolic rate^a^ • Borderline abnormal thyroid function tests^a^	○ *Indirect calorimetry*: reduced resting energy expenditure ○ *Creatine kinase- skeletal muscle isoenzyme (MM)*: raised ○ *Lipid profiles*: raised total and LDL cholesterol ○ *SHBG*: raised or normal ○ *ft4/fT3 ratio*: low or low normal ○ *Reverse T3*: low or normal ○ *IGF-1*: low or normal
Haematological	• Mild anaemia	○ *Full blood count*: low red cell mass or haematocrit with normal MCV and normal B12, folate, reticulocyte count
Neurological & cognitive	• Delayed developmental milestones • Slow, dysarthric speech^a^ • Slow initiation of movement, ataxic gait • Dysdiadochokinesis • Fine and gross motor incoordination (dyspraxia)^a^ • Seizures • ? Autism spectrum disorder	○ *MRI brain*: microcephaly and reduced cerebellar size ○ *Neuropsychological testing*: reduced IQ, low visual, verbal and working memory scores, reduced motor coordination

a Indicates features found in the majority of patients.

b Indicates radiological features found in children only.

**Table 3:** Differential diagnosis of disorders with a high T3, low T4, normal TSH pattern of thyroid function tests.

Disorder	Dyshormonogenesis		Resistance to thyroid hormone α	Allan Herndon Dudley syndrome
Genetic – congenital hypothyroidism	Environmental – iodine deficiency
fT4	Normal or low	Normal or low	Normal or low	Normal or low
fT3	Normal or raised	Raised	Raised	Raised
fT4/fT3 Ratio	Low	Low	Low	Low
TSH	Normal or raised	Normal	Normal	Normal
Reverse T3	Normal	Normal	Normal or low	Low
Thyroglobulin	Raised	Raised	Normal	Normal
Urinary iodine	Normal	Low	Normal	Normal
Clinical features	Goitre	Goitre	Growth retardation	Mental & psychomotor retardation
